# Spread of SARS-CoV-2 Variants on Réunion Island, France, 2021

**DOI:** 10.3201/eid2804.212243

**Published:** 2022-04

**Authors:** Alizé Mercier, David A. Wilkinson, Camille Lebarbenchon, Patrick Mavingui, Luce Yemadje-Menudier

**Affiliations:** Santé Publique France, Saint-Denis, France (A. Mercier, L. Yemadje-Menudier);; Université de La Réunion, Centre National de la Recherche Scientifique, Institut National de la Santé et de la Recherche Médicale, Institut de Recherche pour le Développement, Sainte-Clotilde, France (D.A. Wilkinson, C. Lebarbenchon, P. Mavingui);; Plateforme Technologique du Cyclotron Réunion Océan Indien (CYROI), Sainte-Clotilde (D.A. Wilkinson)

**Keywords:** COVID-19, SARS-CoV-2, severe acute respiratory syndrome coronavirus 2, viruses, respiratory infections, zoonoses, epidemiology, sequencing, variants, France, Réunion Island, *Suggested citation for this article*: Mercier A, Wilkinson DA, Lebarbenchon C, Mavingui P, Yemadje-Menudier L. Spread of SARS-CoV-2 variants on Réunion Island, France, 2021. Emerg Infect Dis. 2022 Apr [*date cited*]. https://doi.org/10.3201/eid2804.212243

## Abstract

In January 2021, after detection of severe acute respiratory syndrome coronavirus 2 (SARS-CoV-2) variants, genomic surveillance was established on Réunion Island to track the introduction and spread of SARS-CoV-2 lineages and variants of concern. This system identified 22 SARS-CoV-2 lineages, 71% of which were attributed to the Beta variant

Coronavirus disease (COVID-19) is a respiratory illness caused by severe acute respiratory syndrome coronavirus 2 (SARS-CoV-2). On Réunion Island, an overseas department of France located in the Indian Ocean, the first cases of COVID-19 were detected on March 11, 2020, in a group of travelers (D.A. Wilkinson et al., unpub. data, https://doi.org/10.1101/2021.01.21.21249623). In response, a regional epidemiologic surveillance focusing on contact tracing and early detection of clusters was conducted. After several months of imported cases and sporadic autochtonous cases, a sharp increase in locally acquired infections was recorded in August 2020, after the return of many Réunion Island residents from travel abroad, primarily mainland France, where the incidence rate was high. The virus subsequently spread throughout the island.

In January 2021, after SARS-CoV-2 variants were detected, genomic surveillance was established to track the introduction and spread of SARS-CoV-2 lineages on the island. During January–June 2021, we generated a total of 1,528 genome sequences with >90% coverage using the ARTIC protocol (https://artic.network/ncov-2019/ncov2019-bioinformatics-sop.html) and nanopore technology (MinION; Oxford Nanopore Technologies, https://nanoporetech.com). This collection represents 8.3% of all COVID-19 cases on Réunion Island during that period (n = 18,409). Sample selection was pseudo-random; a small proportion of cases was prioritized for sequencing because of atypical epidemiologic or clinical characteristics. Pangolin lineages were assigned to all genomes using Pangolin version 1.2.88 (https://github.com/cov-lineages/pango-designation/releases/tag/v1.2.88).

We present the main findings of genomic surveillance from weeks 1–22, 2021 (January 4–June 6, 2021). We focused on the evolution of the weekly proportions of the 8 most frequent SARS-CoV-2 variants and examined the correlation between the weekly number of confirmed cases and the proportion of sequences identified as Beta variant (B.1.351). We extracted lineage distributions in other islands of the Indian Ocean and South Africa from the GISAID database (http://www.gisaid.org) to investigate the origins of the Beta variant sublineages.

We identified 22 SARS-CoV-2 lineages, 71% of which were attributed to the Beta variant (sublineages B.1.351 and B.1.351.2) (Table). On the basis of available data in the GISAID database, lineage B.1.622 seems to be specific to Réunion Island; no other sequence had been reported elsewhere.

**Table:** Observed lineages of severe acute respiratory syndrome coronavirus 2, Réunion, France, 2021

Pangolin lineage	No. genomes
B.1.351.2 (Beta, sub-lineage 2)	716
B.1.351 (Beta, sub-lineage 0)	361
B.1.177	154
B.1.622	71
B.1.1.7 (Alpha)	65
B.1.160	55
B.1.160.18	36
B.1.1.353	18
B.1.617.2 (Delta)	14
B.1.438.2	10
B.1.525 (Eta)	8
B.1.416.1	5
B.1.177.24	3
B.1	3
B.1.177.37	2
B.1.1	1
B.1.1.241	1
B.1.160.27	1
B.1.177.81	1
B.1.221	1
B.1.428.2	1
P.2	1
Total	1,528

The Beta variant was first detected on Réunion Island during the first week of January 2021, although it may have been introduced before its detection by full-genome sequencing. During the first 6 weeks of 2021, lineages known to have high levels of circulation in Europe (e.g., B.1.160, B.1.177) represented most sequenced genomes (Figure). This finding highlights the strong effect of air travel on COVID-19 dynamics on an island such as Réunion (*1*; D.A. Wilkinson et al., unpub. data).

**Figure:**
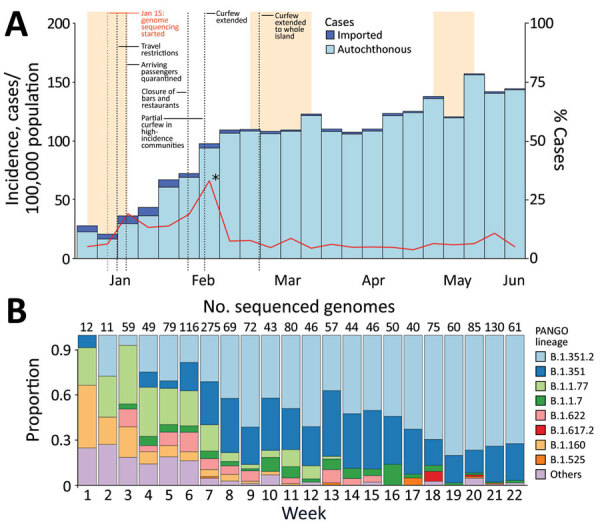
A) Epidemic curve of severe acute respiratory syndrome coronavirus 2cases detected in Réunion, France by week of sampling, weeks 1–22, 2021. Orange bars correspond to school holidays. B) Distribution of severe acute respiratory syndrome coronavirus 2 lineages identified in Réunion, France. Weekly number of sequenced genomes appears above the relevant bar.

Since mid-February 2021 (week 7 of 2021), the Beta variant has become dominant on Réunion Island, despite low-level circulation of the Alpha variant, another variant of concern that was dominant in mainland France and other countries in Europe at that time. We detected a correlation (Spearman ρ = 8.4 × 10^−4^; p<0.001) between the number of COVID-19 cases in January–February 2021 and the number of sequences attributed to the Beta variant, which has been shown to have increased transmissibility (C.A. Pearson et al., unpub. data, https://cmmid.github.io/topics/covid19/sa-novel-variant.html). Several additional factors could explain the dominance of Beta variant; genetic and epidemiologic factors may have contributed to a founder effect, a higher frequency of virus introductions resulting from holiday travels, possible superspreading events, and local and regional contexts (*2*). Indeed, geographic proximity and population movements with Mayotte, another overseas department of France, and Comoros link Réunion Island to South Africa, where Beta variant was first reported (*3*).

We detected 2 sublineages of Beta variant, B.1.351 and B.1.351.2. Sublineage B.1.351.2 accounted for 3-fold more cases than B.1.351. It was detected concurrently in Mayotte, Comoros, and Réunion Island. This finding, coupled with information from GISAID, suggests that lineage B.1.351.2 was imported to Comoros and Mayotte from South Africa and could have been introduced to Réunion Island from Mayotte (*4*) (Appendix 1). This possible introduction from Mayotte is supported by the flow of travelers between the 2 departments and the notable peak in COVID-19 cases that occurred in Mayotte during weeks 1–11, mainly caused by the Beta variant (*5*). However, analysis of the origin of lineages is strongly affected by each location’s capacity to sequence and report genomes in GISAID, which renders comparison between different locations difficult (*4*).

Our study provides valuable insights into the interactions between SARS-CoV-2 lineages on Réunion Island, which represents a closed system with controlled entries, especially when travel restrictions are in place. Additional research on genomic epidemiology and the effect of air travel can further improve understanding of why some variants become dominant over others, particularly in insular contexts. The future of genomic surveillance on Réunion Island will focus on mutation screening to increase reactivity, combined with real-time sequencing, as a robust approach to track the spread of emerging SARS-CoV-2 variants of concern and to inform public health actions (*6*,*7*).

Appendix 1 Additional information from study of the spread of severe acute respiratory syndrome coronavirus 2 variants on the island of Réunion, France 2021.

Appendix 2 Information about severe acute respiratory syndrome coronavirus 2 sequences obtained from GISAID in study of variants on the island of Réunion, France, 2021.
