# Supplementary material for: Spread of SARS-CoV-2 Variants on Réunion Island, France, 2021
Source: Emerg Infect Dis. 2022 Apr;28(4):895–8. doi: 10.3201/eid2804.212243 (PMC8962908; doi:10.3201/eid2804.212243)
Supplement: Appendix 1 — Additional information from study of the spread of severe acute respiratory syndrome coronavirus 2 variants on the island of Réunion, France 2021. [file 21-2243-Techapp-s1.pdf]

Article DOI: https://doi.org/10.3201/eid2804.212243

Spread of SARS-CoV-2 Variants on Réunion Island, France, 2021

Appendix 1

GISAID Sequences

We searched in the GISAID database for sequences collected over the same time period as our study (January 4–June 6, 2021). The amount of sequencing data available from the different countries in the region was highly variable. We obtained 705 sequences from Mayotte, where B.1.351.2 accounted for 49% of all submitted sequences and was more frequent than B.1.351. Only 6 sequences were available from the neighboring islands of the Comoros archipelago, of which all were B.1.351.2. There were 27 sequences available from the Seychelles, most of which were B.1.351. Réunion's closest neighboring island, Mauritius, had 153 sequences deposited over the same study period; none were from lineage B.1.351.2. Three isolates were associated with the Beta variant, which suggested that Mauritius and Réunion have highly contrasting epidemiologic circulation of SARS-CoV-2 variants. The amount of data from Madagascar was also limited considering its size and population; 569 sequences were available for the study period. B.1.351.2 lineage only became prevalent among GISAID-deposited genomes from Madagascar in week 07-2021, after its emergence and circulation in other islands of the southwestern Indian Ocean.

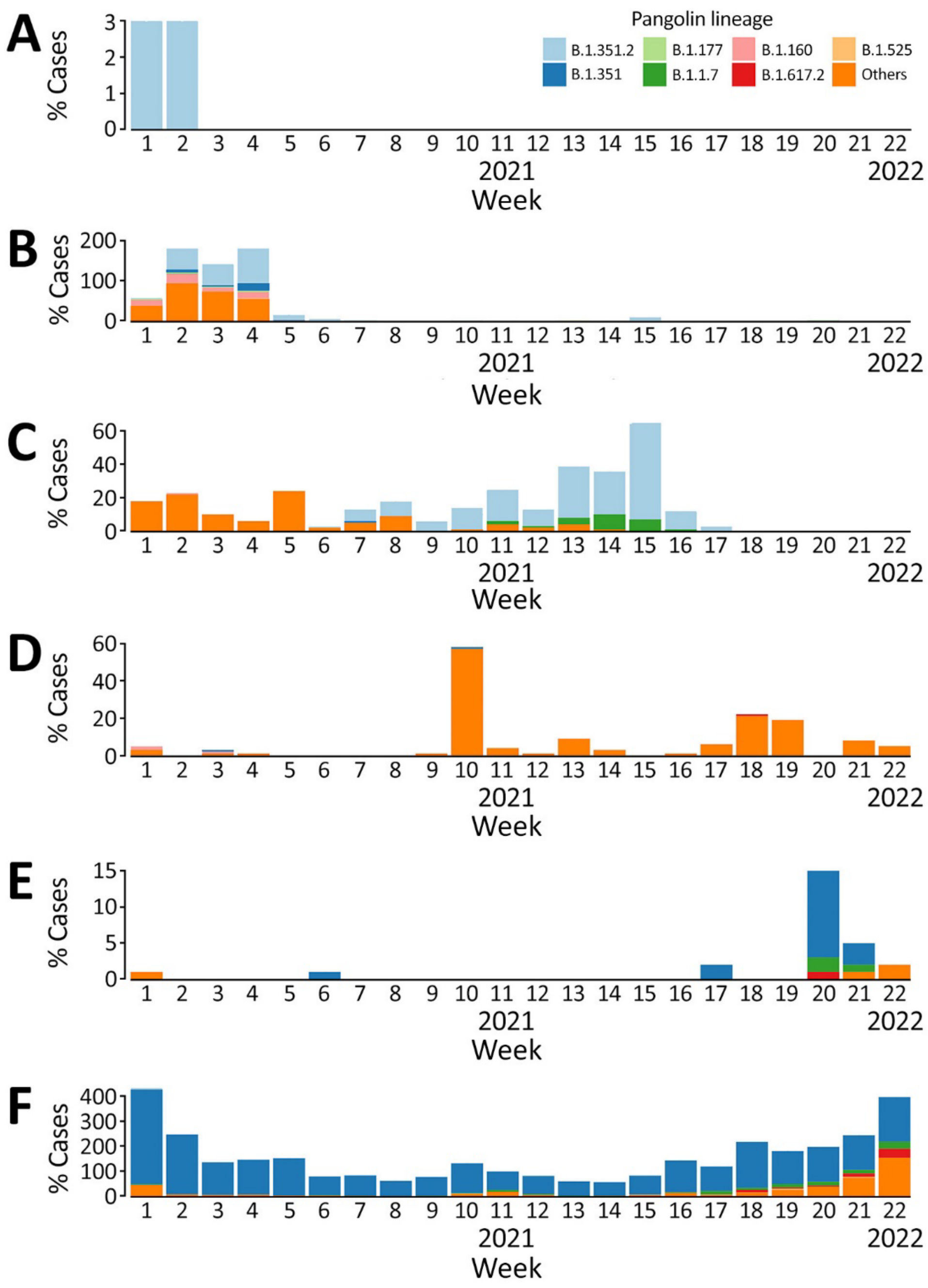

Appendix Figure. Circulation of SARS-CoV-2 lineages in islands of the southwestern Indian Ocean and South Africa, weeks 01–22, 2021. A) Comoros. B) Mayotte. C) Madagascar. D) Mauritius. E) Seychelles, F) South Africa.
